# Sustained Enhancement of Lateral Inhibitory Circuit Maintains Cross Modal Cortical Reorganization

**DOI:** 10.1371/journal.pone.0149068

**Published:** 2016-02-10

**Authors:** Waki Nakajima, Susumu Jitsuki, Akane Sano, Takuya Takahashi

**Affiliations:** Department of Physiology, Yokohama City University Graduate School of Medicine, Yokohama, Japan; Bilkent University, TURKEY

## Abstract

Deprivation of one modality can lead to the improvement of other intact modalities. We have previously reported that visual deprivation drives AMPA receptors into synapses from layer4 to 2/3 in the barrel cortex and sharpens functional whisker-barrel map at layer2/3 2 days after the beginning of visual deprivation. Enhanced excitatory synaptic transmission at layer4-2/3 synapses is transient and returns to the base line level a week after the beginning of visual deprivation. Here we found that sharpened whisker-barrel function is maintained at least for a week in visually deprived animals. While increased AMPA receptor-mediated synaptic transmission at layer4-2/3 synapses dropped to the base line a week after the beginning of visual deprivation, lateral inhibitory synaptic transmission onto the neighboring barrel was kept strengthened for a week of visually deprived animals. Thus, transient strengthening of excitatory synapses at layer4-2/3 in the barrel cortex could trigger the enhancement of inhibitory inputs to neighboring barrel, and sustained lateral inhibition can maintain the sharpening of whisker-barrel map in visually deprived animals.

## Introduction

Loss of one sensory system could enhance the function of other remaining modalities. Although this type of plasticity, cross modal plasticity, is well known, molecular and cellular basis underlying it is still poorly understood [1–4]. We have previously reported that visual deprivation drives AMPA receptors into layer4-2/3 pyramidal synapses in the juvenile barrel cortex and leads to the improvement of whisker-barrel function [5]. This process is mediated by the increase of the extracellular serotonin in the barrel cortex [5]. Visual deprivation merely transiently strengthens excitatory synapse mediated by synaptic trafficking of AMPA receptor [5], a well characterized cellular and molecular basis of experience dependent neural plasticity [6–20]. Although visual deprivation increases AMPA receptor-mediated synaptic transmission at layer4-2/3 pyramidal synapses 2 days after the beginning of visual deprivation, the enhanced excitatory transmission returns to the basal level a week after the beginning of visual deprivation [5].

Here we found that visual deprivation-induced sharpening of whisker-barrel map is sustained until a week after the beginning of visual deprivation even when enhanced excitatory synaptic transmission at layer4-2/3 synapses returned to the basal level. Interestingly, visual deprivation also strengthened inhibitory synaptic input onto neighboring barrel, and this enhanced inhibitory synaptic transmission at horizontal pathway sustained until a week after the beginning of visual deprivation. Thus, visual deprivation-induced enhancement of excitatory synaptic transmission at layer4-2/3 synapses could trigger the strengthening of horizontal inhibitory synapses, and sustained lateral inhibition could maintain sharpness of whisker barrel map in visually deprived animals.

## Methods

### Ethics statement

All experiments were conducted according to the Guide for the Care and Use of Laboratory Animals (Japan Neuroscience Society) and the Guide for the Yokohama City University. All the animal experiments were approved by the Animal Care and Use Committee of Yokohama City University (authorization number: F-A-14-024). All surgical procedures were performed under anesthesia, and every effort was made to minimize suffering during the surgical procedures and post-surgery recovery.

### Animals

Male juvenile Long-Evans rats (postnatal day 21–28) were housed on a 12-hour light/dark cycle with *ad libitum* access to water and food. Visual deprivation was introduced by using binocular suturing under anesthesia with isoflurane-oxygen mixture. Procedures were performed in strict compliance with the animal use and care guidelines of Yokohama City University.

### Drug treatment

Rats were deeply anesthetized with an isoflurane-oxygen mixture. The skin overlying the skull was cut and gently pushed to the side. A craniotomy (1-mm diameter) was opened above the right barrel cortex (2 mm posterior from bregma; 4.5 mm lateral from midline) with a dental drill. 2mM Volinanserin or Saline (Vehicle) was pressure injected through a pulled-glass capillary (Narishige) into the barrel cortex. To visualize drug-injected area, drugs were mixed with Alexa594 (Thermo Fisher Scientific). After injection, the skin was repositioned with cyanoacrylate glue and sutured both eyes. Rats were kept on a heating pad during the procedures, and returned to their home cages after regaining movement.

### Electrophysiology

Rats were anesthetized with an isoflurane-oxygen mixture. The Brains were quickly transferred into gassed (95% O_2_ and 5% CO_2_) ice-cold dissection buffer as described previously [5]. Coronal brain slices were cut (350 μm, Leica VT1000) in dissection buffer. Slices were then incubated in artificial cerebrospinal fluid (ACSF) containing 118 mM NaCl, 2.5 mM KCl, 4 mM CaCl_2_, 4 mM MgCl_2_, 26 mM NaHCO_3_, 1 mM NaH_2_PO_4_, 10 mM glucose [6].

For recordings of AMPA/NMDA ratio or IPSC/EPSC ratios, Whole-cell recordings were obtained from layer 2/3 pyramidal neurons of the barrel cortex using patch recording pipettes (3–7 MΩ) filled with intracellular solution as described previously [5, 14, 21]. Bipolar tungsten stimulating electrodes were placed in layer 4 (vertical input) or layer 2/3 of adjacent side ~200–300 μm from recorded cells. AMPA/NMDA ratios were calculated as the ratio of the peak current at -60 mV to the current at +40 mV 50 ms after stimulus onset (40–50 traces averaged for each holding potential) with 4 μM 2-chloroadenosine and 0.1mM picrotoxin. To avoid the stimulation of same sets of synapses for the recording from distinct neurons, we picked up neurons from different barrel columns. IPSC/EPSC ratios were calculated as the ratio of the peak current at 0 mV to the current at -60 mV with 4 μM 2-chloroadenosine and 0.1 mM D,L-APV.

For recording of asynchronous mIPSC in lateral Input to pyramidal neurons, slices were incubated with ACSF containing 118 mM NaCl, 2.5 mM KCl, 4 mM SrCl_2_, 1 mM MgCl_2_, 26 mM NaHCO_3_, 1 mM NaH_2_PO_4_, 10 mM glucose in recording chamger. Patch recording pipettes (3–7 MΩ) were filled with 100 mM CsMeSO_3_, 55 mM CsCl, 10 mM HEPES, 2 mM BAPTA, 4 mM Na_2_-ATP, 0.4 mM Na-GTP, 10 mM Na-Phosphocreatine, 1 mM MgCl_2_ (pH adjusted to 7.0 with CsOH) [22]. Bipolar tungsten stimulating electrodes were placed in layer 2/3 of adjacent side ~200–300 μm from recorded cells. The mIPSC was recorded at -75 mV with 20 μM CNQX and 0.1 mM D,L-APV.

For recording of asynchronous mEPSC in inhibitory neurons, slices were incubated with ACSF containing 118 mM NaCl, 2.5 mM KCl, 1 mM MgCl_2_, 26 mM NaHCO_3_, 1 mM NaH_2_PO_4_, 10 mM glucose in recording chamber. To distinguish the type of interneuron, Current-clamp recording were obtained from layer 2/3 neurons with patch recording pipettes (3–7 MΩ) were filled with 130mM C_6_H_11_KO_7_, 2mM KCl, 2mM MgCl_2_, 3mM ATP, 0.3mM GTP, 10mM HEPES, 20mM biocytin (pH adjusted to 7.0 with KOH). Depolarizing current steps were introduced into each cell and then record firing responses. After current-clamp recordings, 4mM SrCl_2_ were added into recording ACSF. Asynchronous mEPSC was recorded at -60mV with stimulation of bipolar tungsten electrodes placed in layer 4. After recording, slices were histologically analyzed. Data were analyzed using Clampfit10.2 (Molecular Devices) or Mini analysis program 6.0.7 (Synaptosoft).

### Histology

Slices containing biocytin-loaded cells were fixed by 4% paraformaldehyde overnight at 4°C, and were cut at thickness of 50μm. Slices were incubated with 1% Texas red streptavidin (Vector Labolatories) in 0.1M Tris HCL-buffered saline with 0.5% Triton X-100 for 1 hour at room temperature. Texas red-labeled neurons were imaged with fluorescent microscopy (Olympus FV-10).

### In vivo recording

Rats were anesthetized with urethane (1.25 mg/kg). A craniotomy was opened above the right barrel cortex as described above. The barrel column was randomly selected for recording in each animal. Tungsten electrodes (California Fine Wire Co.) were inserted 200–300μm below the pia. The detailed procedure for this recording has been described previously [6]. The number of spikes from 5 ms to 50 ms after whisker deflection was used as a measure of the response. Then, the ratio of spike frequency in response to surround whiskers normalized to that of principal whisker was calculated.

### Statistics

All data are presented as mean ± SEM. All statistics were analyzed using SPSS software(SPSS 22.0; IBM). Data were analyzed by student’s *t*-test or one-way ANOVA. When comparing more than two groups, ANOVA followed by Bonferroni post-hoc analyses or Fisher's Least Significant Difference was used. P < 0.05 was considered statistically significant.

## Results

### Visual deprivation-induced strengthening of excitatory synaptic transmission at layer4-2/3 synapses is transient

**Fig 1:**
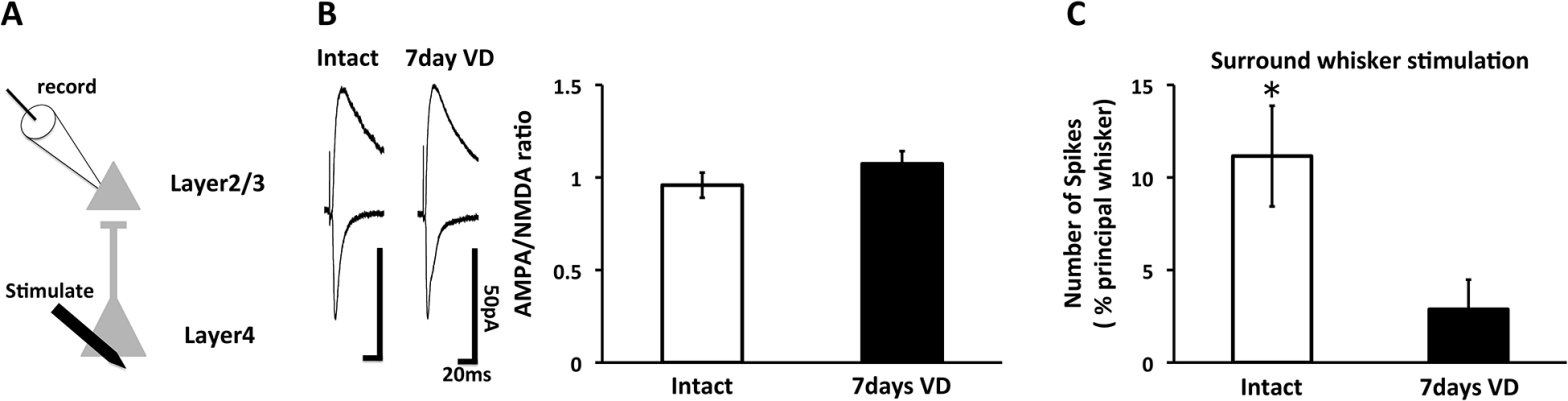
Visual deprivation-induced enhancement of AMPA receptor-mediated currents at layer4-2/3 synapses of the juvenile barrel cortex lasts for a week. (A) Schematic representation of electrophysiological experiment. Excitatory synaptic responses of layer 4-2/3 synapses were recorded in this experiment. (B) (Left) Synaptic responses at layer 4-2/3 synapses in the barrel cortex of intact rats (Intact) or rats with 7days visual deprivation (7days VD). (Right) Mean ratio of AMPAR-mediated currents to NMDA receptor-mediated currents (A/N ratio). Note no difference of A/N ratio between Intact and 7days VD. (Intact: n = 9, 7days VD: n = 10). (C) Responses (spike frequency) in layer 2/3 neurons to deflection of first-order surrounding (S1) whiskers normalized to the response for the principal whisker. 7days VD showed lower values in S1 than Intact, indicating that the whisker-barrel map was remained sharpened in 7days VD rats (Intact: n = 10, 7days VD: n = 8. *, p < 0.05, Student's *t*-test. Error bars, ±SEM).

To examine how long the visual deprivation-induced strengthening of AMPA receptor-mediated synaptic transmission at layer4-2/3 synapses in the barrel cortex lasts, we sutured both eyes at P21, prepared acute brain slices at P28 (7 days after visual deprivation) and performed whole cell recordings. Despite the increase of AMPA receptor-mediated synaptic transmission/NMDA receptor-mediated synaptic transmission (AMPA/NMDA ratio) at P23 [5], we detected the comparable A/N ratio in 7 days visually-deprived rats as was observed in intact rats (Fig 1A and 1B). This suggest that visual deprivation-induced strengthening of AMPA receptor-mediated synaptic transmission is transient and returned to base line 7 days after the eye suture.

### Visual deprivation-induced sharpening of functional whisker-barrel map maintains 7 days after the beginning of visual deprivation

We have previously reported that visual deprivation sharpens functional whisker-barrel map at layer2/3 of the barrel cortex 2 days after visual deprivation [5]. We wondered whether the visual deprivation-induced sharpening of functional whisker-barrel map maintains for a week after visual deprivation when excitatory synaptic transmission at layer4-2/3 synapses returns to the base line. To test this possibility, we examined receptive fields of layer 2/3 of the barrel cortex in the absence or presence of visual deprivation. 7 days after visual deprivation, single-unit *in vivo* recording was performed in anaesthetized rats to assess the functional whisker-barrel map. We measured the number of spikes evoked in response to deflections of multiple single whiskers. Whiskers that exhibited the largest response were defined as principal whiskers. We found that the relative response of surrounding whiskers to the response evoked by stimulation of the principal whiskers was lower in rats exposed to visual deprivation than in control rats even 7 days after the beginning of visual deprivation, indicating that sharpened functional receptive fields of layer 2/3 in the barrel cortex were maintained for a week after visual loss (Fig 1C).

### Visual deprivation strengthens inhibitory synapses at horizontal synapses in the barrel cortex

**Fig 2:**
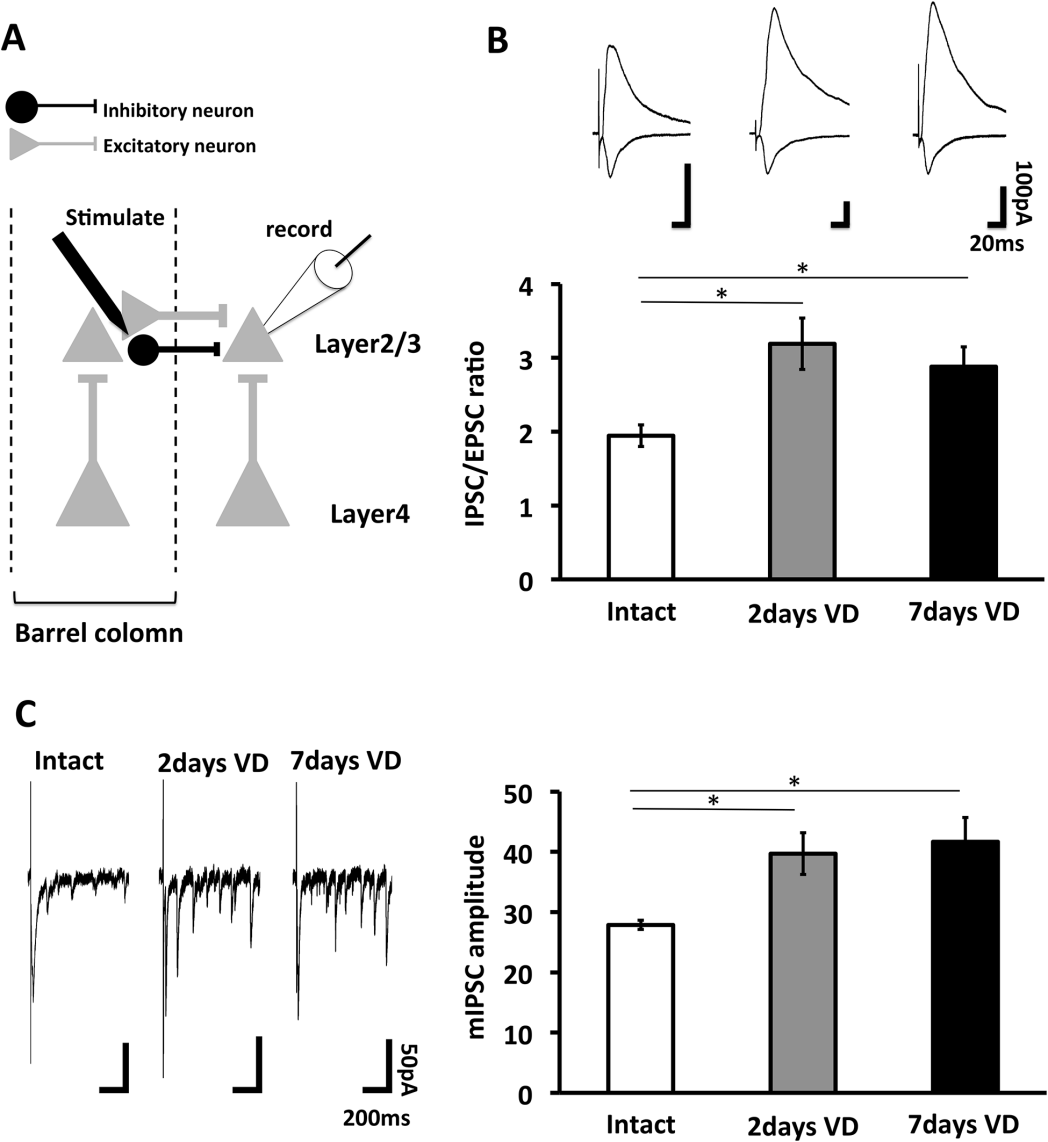
Visual deprivation increases inhibitory lateral input in the juvenile barrel cortex. (A) Schematic representation of electrophysiological experiment. Excitatory and inhibitory synaptic responses of layer 2/3-2/3 synapses (lateral pathway) were recorded in this experiment. (B) (Top) Synaptic responses at layer 2/3-2/3 synapses in the barrel cortex of intact rats (Intact), rats with 2days VD (2days VD) or 7daysVD. (Bottom) Mean ratio of IPSC (holding potential at 0mV with APV) to EPSC (holding potential at -60mV) (IPSC/EPSC ratio). IPSC/EPSC ratio of 2days VD and 7days VD were significantly higher than Intact (Intact: n = 10, 2days VD: n = 11, 7days VD: n = 12. *, p < 0.05, ANOVA followed by Bonferroni post-hoc analyses. Error bars, ±SEM). (C) (Left) Evoked miniature IPSC (mIPSC) responses at layer 2/3-2/3 synapses in the barrel cortex of Intact, 2days VD or 7days VD (holding potential at -75mV with CNQX). (Right) Mean amplitude of evoked mIPSCs. 2days VD and 7days VD exhibited the increased amplitude of evoked mIPSCs compared to Intact (Intact: n = 7, 2days VD: n = 8, 7days VD: n = 8. *, p < 0.05, ANOVA followed by Bonferroni post-hoc analyses. Error bars, ±SEM).

In our previous report, we found that visual deprivation strengthens excitatory vertical layer4-layer-2/3 pyramidal synapses but not layer2/3-layer2/3 horizontal synapses 2 days after the beginning of visual deprivation [5], and considered that the enhanced strength at layer4-2/3 synapses compared to layer2/3-2/3 horizontal synapses could result in the sharpening of functional whisker-barrel map. However, while once enhanced excitatory layer4-2/3 synapses in the barrel cortex of visually deprived animals returns to base line 7 days after visual loss, sharpened functional whisker-barrel map was still maintained. How is the sharpened functional whisker-barrel map maintained? To this end, we hypothesized that lateral inhibitory input to neighboring barrel could be strengthened by visual deprivation and enhanced lateral inhibition could be maintained for a week. In order to examine this hypothesis, we sutured both eyes at P21 and prepared acute brain slices at P23 (2 days after visual deprivation) and at P28 (7 days after visual deprivation). We examined inhibitory postsynaptic currents (IPSCs) at layer2/3 pyramidal neurons by stimulating neighboring barrel with whole cell recordings. We first measured ratio of IPSCs to EPSCs (IPSC/EPSC ratio) at these synapses. We found the increase of IPSC/EPSC ratio at horizontal synapses at P23 and P28, indicating that visual deprivation induces enhancement of lateral inhibitory inputs and the increase of lateral inhibition continues until 7 days after the beginning of visual deprivation (Fig 2A and 2B). To further confirm this, we examined evoked miniature IPSCs (mIPSCs) at horizontal synapses. We found that amplitude of evoked mIPSCs was increased 2 days after visual loss and remained enhanced 7days after the beginning of VD (Fig 2C). These results suggest that VD-induced strengthening of lateral inhibition remains maintained for a week and could result in the sustained sharpening of functional whisker-barrel map.

**Fig 3:**
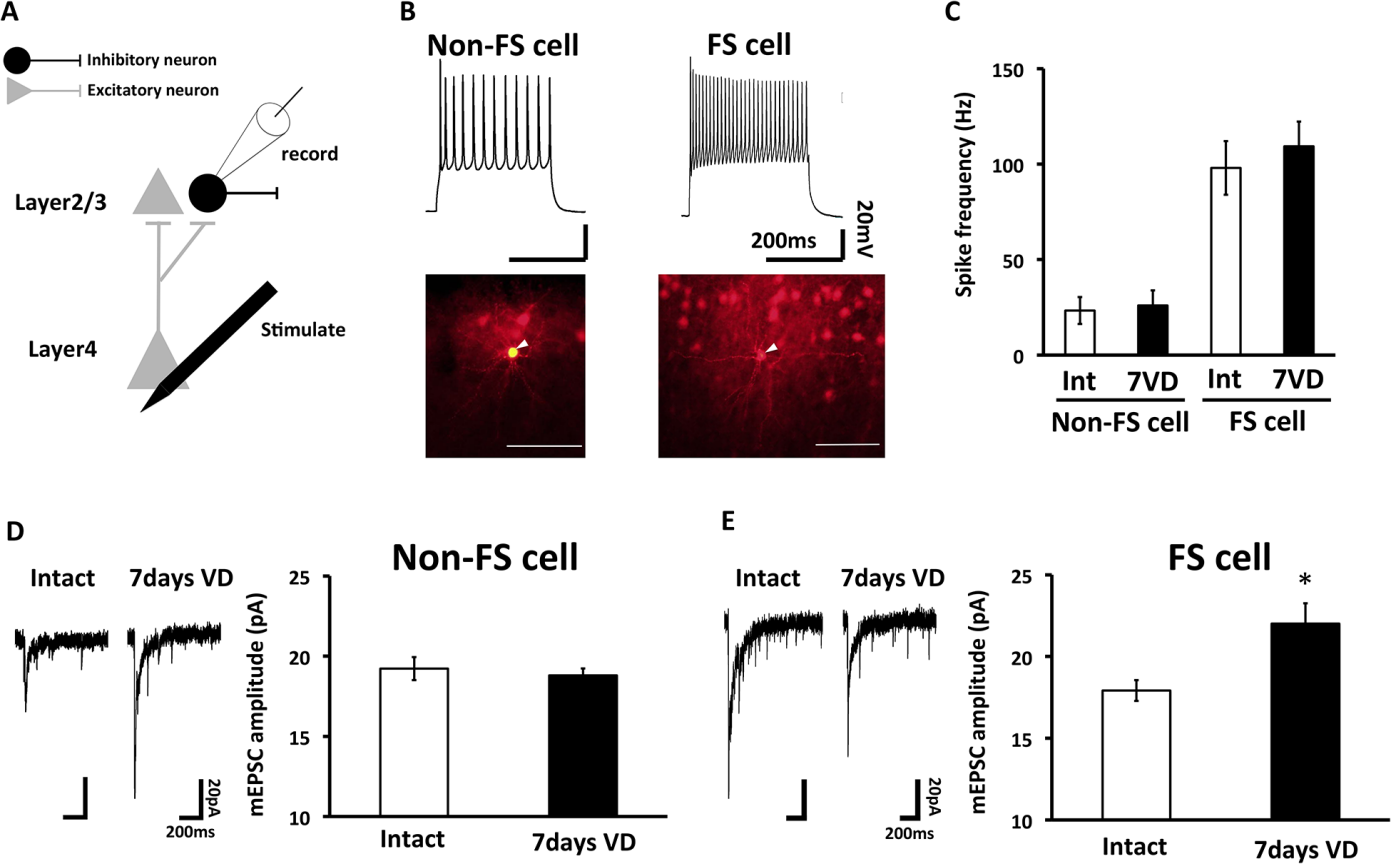
Visual deprivation augments excitatory input onto fast-spiking but not non-fast-spiking neurons. (A) Schematic representation of electrophysiological experiment. Current clamp recordings were obtained from interneurons and recorded the firing pattern. After current clamp recordings, SrCl_2_ was applied into ACSF. Evoked miniature EPSCs (mEPSCs) were recorded with the voltage clamp mode at -60 mV. (B) (Top) Example of the firing patterns in non fast-spiking cells and fast-spiking cells. (Bottom) Example of the morphology of non fast-spiking and fast-spiking cell. Non fast-spiking cells have local axonal arborizations and fast-spiking cells have expansive axonal arborizations (Arrow head shows soma. Scale bars indicate 100μm). (C) Mean firing frequency in non fast-spiking cells and fast-spiking cells of intact rats (Int) or rats with 7days VD (7VD). (D) (Left) Synaptic responses at layer 4-2/3 synapses in non fast-spiking cells of intact rats (Intact) or rats with 7days VD (7days VD). (Right) Mean amplitude of evoked mEPSC (holding potential at -60mV). Note no difference in amplitude of evoked mEPSC between Intact and 7days VD (Intact: n = 6, 7days VD: n = 5). (E) (Left) Synaptic responses at layer 4-2/3 synapses in fast-spiking cells of Intact or 7days VD. (Right) Mean amplitude of evoked mEPSC. 7days VD exhibited the increased amplitude of evoked mEPSCs compared to Intact (Intact: n = 5, 7days VD: n = 6. *, p < 0.05, Student's *t*-test. Error bars, ±SEM).

What is a mechanism underlying the increase of lateral inhibitory input by visual deprivation. We hypothesized that vertical excitatory input onto interneurons were strengthened by visual deprivation. To test this, we performed whole cell recordings from interneurons at layer 2/3 of animals with 7 days-visual deprivation. We distinguished cell types of interneurons from the firing pattern and the morphology (analyzed by intracellular injection of biocytin from the patch pipet). Interestingly, vertical excitatory inputs (evoked mEPSCs) onto fast spiking interneurons which extends axons to the neighboring barrel [23] (Fig 3A, 3B and 3C) were strengthened compared to non-fast spiking interneurons (Fig 3D and 3E). These results suggest visual deprivation-induced strengthening of input onto interneurons which may inhibit pyramidal neurons in the neighboring barrel.

### Serotonin 5HT2A receptor mediates enhancement of lateral inhibition

**Fig 4:**
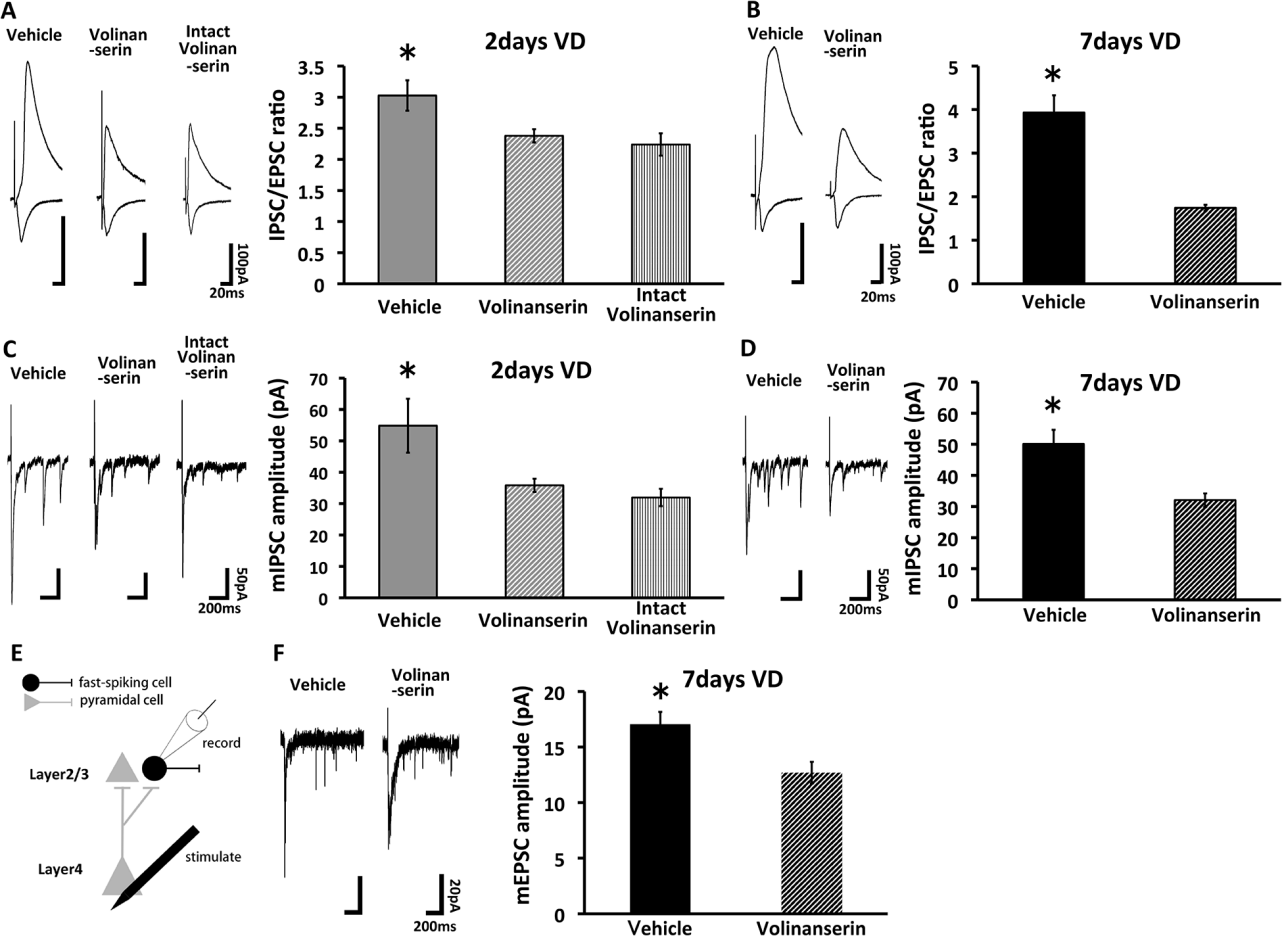
5HT2A receptor mediates visual deprivation-induced enhancement of lateral inhibitory pathways. (A) (Left) Synaptic responses at layer 2/3-2/3 synapses in the barrel cortex of vehicle-treated rats with 2days VD (Vehicle), Volinanserin (5HT2A receptor antagonist)-treated rats with 2days VD (Volinanserin) or Volinanserin-treated intact rats (Intact Volinanserin). (Right) Mean IPSC/EPSC ratio. Vehicle exhibited the larger IPSC/EPSC ratio compared to Volinanserin and Intact Volinanserin (Vehicle: n = 9, Volinanserin: n = 8, Intact Volinanserin: n = 8. *, p < 0.05, ANOVA with Fisher's Least Significant Difference test. Error bars, ±SEM). (B) (Left) Synaptic responses at layer 2/3-2/3 synapses in the barrel cortex of vehicle-treated rats with 7days VD (Vehicle) or Volinanserin -treated rats with 7days VD (Volinanserin). (Right) Mean IPSC/EPSC ratio. Vehicle exhibited the larger IPSC/EPSC ratio compared to Volinanserin (Vehicle: n = 10, 7days Volinanserin: n = 7. *, p < 0.05, Student's *t*-test. Error bars, ∓SEM). (C) (Left) Evoked miniature IPSC (mIPSC) responses at layer 2/3-2/3 synapses in the barrel cortex of vehicle-treated rats with 2days VD (Vehicle), Volinanserin -treated rats with 2days VD (Volinanserin) or Volinanserin-treated intact rats (Intact Volinanserin). (holding potential at -75mV with CNQX). (Right) Mean amplitude of evoked mIPSCs. Vehicle exhibited the larger amplitude of evoked mIPSCs compared to Volinanserin and Intact Volinanserin (Vehicle: n = 6, Volinanserin: n = 7, Intact Volinanserin: n = 7. *, p < 0.05, ANOVA followed by Bonferroni post-hoc analyses. Error bars, ∓SEM). (D) (Left) Evoked mIPSC responses at layer 2/3-2/3 synapses in the barrel cortex of vehicle-treated rats with 7days VD (Vehicle) or Volinanserin-treated rats with 7days VD (Volinanserin). (Right) Mean amplitude of evoked mIPSCs. Vehicle exhibited the larger amplitude of evoked mIPSCs compared to Volinanserin (Vehicle: n = 8, Volinanserin: n = 7. *, p < 0.05, Student's *t*-test. Error bars, ±SEM). (E) Schematic representation of electrophysiological experiment. Current clamp recordings were obtained from fast-spiking cells and recorded the firing pattern. After current clamp recordings, SrCl_2_ was applied into ACSF. Evoked miniature EPSCs (mEPSCs) were recorded with the voltage clamp mode at -60 mV. (F) (Left) Synaptic responses at layer 4-2/3 synapses of fast-spiking cells in 7days VD rats treated with Vehicle or Volinanserin. (Right) Mean amplitude of evoked mEPSCs. 7days VD with vehicle treatment (Vehicle) exhibited the larger amplitude of evoked mEPSCs compared to Volinanserin (Vehicle: n = 5, Volinanserin: n = 6. *, p < 0.05, Student's *t*-test. Error bars, ±SEM).

To examine if serotonin signaling mediate the strengthening of the lateral inhibitory input, we performed blockade of 5HT2A signaling pharmacologically. We sutured both eyes at P21 and injected 5HT2A receptor antagonists (Volinanserin) [24, 25] into barrel cortex soon after the eye closure. 2 days or 7days later, IPSC/EPSC ratio at these synapses were recorded by stimulating neighboring barrel with whole cell recordings. We found the increase of IPSC/EPSC ratio at horizontal synapses were decreased in Volinanserin-treated animals with visual deprivation at 2 days and 7 days (Fig 4A and 4B), indicating that enhanced inhibitory layer2/3-2/3 synapses in the barrel cortex of visually-deprived animals is mediated by the activation of serotonin signaling. To further confirm this, we examined evoked miniature IPSCs (mIPSCs) at horizontal synapses. We found that the increase of the amplitude of the evoked mIPSCs at 2 days and 7 days after visual loss was blocked in the presence of Volinanserin (Fig 4C and 4D). As a control experiment, we examined the effect of Volinanserin on the lateral pathways (layer2/3-layer2/3 pyramidal neurons) of the barrel cortex of visually undeprived animals. We found no effects on the baseline IPSCs by the application of Volinanserin (IPSC/EPSC ratio and the amplitude of the evoked mEPSC) (Figs 2B, 2C, 4A and 4C). Further, we found that the application of Volinanserin prevented visual-deprivation induced strengthening of AMPA receptor-mediated synaptic currents (evoked mEPSC) at layer4-2/3 synapses onto fast-spiking interneurons (Fig 4E and 4F). These results indicate that the enhancement of lateral inhibitory input induced by visual deprivation is mediated by the activation of 5HT2A.

## Discussion

Despite the transient increase of excitatory synaptic input from layer 4 to layer 2/3 pyramidal neurons in the barrel cortex of juvenile rats with visual deprivation (it only lasted for a few days), we observed sustained strengthening of excitatory input from layer 4 to layer 2/3 fast-spiking interneurons for at least a week and this could result in the maintenance of lateral inhibition and the sharpened functional whisker-barrel map at layer 2/3. What makes this cell type specificity of the maintenance of the enhanced excitatory input remains to be elucidated. It may be that the effect of visual deprivation is delayed in onset in the fast-spiking neurons compared to the pyramidal neurons.

The plasma half-life of volinanserin is approximately 6.6 hours in human [26]. In addition, we injected this drug into the brain only once when we sutured both eyes. This suggests that the activation of 5HT2A is crucial for the initiation of the plasticity.

We have recently reported that hippocampus dependent contextual fear learning drives AMPA receptors into hippocampal CA3-CA1 neurons and results in the increase of excitatory input to pyramidal neuron [14]. However, this increase is transient and diminishes by 24 hours after conditioning and return to the base line level [14]. On the other hand, contextual fear learning increase the inhibitory inputs and this lasts at least 24 hours after conditioning [14]. In this study, we also observed that inhibition lasts longer than excitation in the barrel cortex of visually deprived animals. Plasticity-inducing experience increase the net excitation transiently, and this could result in the enhancement of inhibition which last for a long time.

A previous paper showed that evoked inhibition from all layers to layser2/3 neurons is decreased in the auditory cortex after the dark exposure [27]. Here we found that the strengthening of the vertical inputs to fast-spiking inhibitory neurons could result in the increase of lateral inhibitory input to layer2/3 pyramidal neurons from the neighboring barrel in the barrel cortex of visually deprived animals (Fig 2). This could lead to the refinement of the functional circuit in the cortical areas responsible for other modalities of animals with visual deprivation. The discrepancy from the result of Meng et al. [27] could be due to the difference of the brain region analyzed. A recent other study reported that the dark exposure does not increase the inhibitory input to layer 2/3 pyramidal neurons in the auditory cortex [28]. This inhibitory input could be local circuits rather than those from neighboring columns with long distance. While we detected the increase of inhibitory inputs to layer 2/3 pyramidal neurons from the neighboring barrel of visually deprived animals, no increase of inhibitory inputs to layer2/3 pyramidal neurons from non-fast spiking neurons was observed (Fig 3). Taken together, these changes in inhibitory inputs refines cortical circuits after visual deprivation.
